# Clonal relationships between Tph and Tfh cells in patients with SLE and in murine lupus

**DOI:** 10.21203/rs.3.rs-7472890/v1

**Published:** 2025-09-12

**Authors:** Takanori Sasaki, John Sowerby, Yinan Xiao, Runci Wang, Snigdha Samarpita, Tatsuki Abe, Kathryne E. Marks, Alice Horisberger, Yidan Gao, Pui Y. Lee, Yujie Qu, Marc A. Sze, Stephen E. Alves, Marc C. Levesque, Keishi Fujio, Karen H. Costenbader, Peter T. Sage, Deepak A. Rao

**Affiliations:** 1 Division of Rheumatology, Inflammation, Immunity, Brigham and Women’s Hospital and Harvard Medical School, Boston, MA, USA; 2 Transplantation Research Center, Division of Renal Medicine, Brigham and Women’s Hospital and Harvard Medical School, Boston, MA, USA; 3 Department of Allergy and Rheumatology, Graduate School of Medicine, The University of Tokyo, Tokyo, Japan; 4 Division of Immunology, Boston Children’s Hospital and Harvard Medical School, Boston, MA, USA; 5 Merck & Co., Inc., Cambridge, MA, USA

**Keywords:** Systemic lupus erythematosus, T follicular helper cell, T peripheral helper cell, T cell receptor repertoire

## Abstract

Pathologic T cell-B cell interactions drive disease in systemic lupus erythematosus (SLE). The T cells that activate B cell responses include T peripheral helper (Tph) and T follicular helper (Tfh) cells, yet the developmental and clonal relationships between these B cell-helper T cell populations are unclear. Here we use T cell receptor (TCR) profiling to demonstrate substantial clonal overlap between Tph and Tfh cells in the circulation of patients with SLE. Expanded Tph and Tfh cell clones persist over the course of 1 year in patients with a new diagnosis of SLE, and clones are observed to shift both from Tfh to Tph cells and from Tph to Tfh cells over time. High resolution analysis of cells sorted as Tph cells (CXCR5^−^ PD-1^hi^) from SLE patients revealed considerable heterogeneity among these cells and highlighted a subpopulation of cells with transcriptomic features of activated B cell-helper T cells. This cell population, marked by expression of *TOX* and *CXCL13*, was found in both sorted Tph and Tfh cells, and was clonally linked in these two populations. Analysis of B cell-helper T cells in murine pristane-induced lupus demonstrated similar populations of Tph and Tfh cells in both lung and spleen with strong clonal overlap. T cell-specific loss of Bcl6 prevented accumulation of Tfh cells and reduced accumulation of Tph cells in pristane-treated mice, indicating a role for Bcl6 in the survival and expansion of both populations. Together, these observations demonstrate a shared developmental path among pathologically expanded Tph and Tfh cells in SLE. The persistence of expanded Tph and Tfh cells clones over time may explain the lack of stable tolerance induction by immunosuppressive medications or by B cell depletion.

## Introduction

Systemic lupus erythematosus (SLE) is a systemic autoimmune disease that can cause immune-mediated injury to multiple organs (1). Pathologic interactions of T cells and B cells and production of autoantibodies are core features of SLE immunopathology (2). Production of autoantibodies typically requires both autoreactive B cells and help from autoreactive T cells, which express T cell receptors (TCR) capable of recognizing the targeted self-antigens. Analyses of circulating T cells from patients with SLE have demonstrated an increase in circulating CXCR5^+^ PD-1^hi^ T follicular helper (Tfh) cells, as well as a prominent increase in circulating CXCR5^−^ PD-1^hi^ T peripheral helper (Tph) cells (3–7). Both Tfh and Tph cells are considered “B cell-helper T cells,” as they are capable of recruiting and activating B cells through production of the B cell chemoattractant CXCL13 and the cytokine IL-21, which is required to induce B cell differentiation into plasmablasts (PB) and age-associated B cells (ABC)(3, 8, 9). Expansion of circulating Tph and Tfh cells has been observed in multiple SLE patient cohorts, generally with higher frequencies in patients with active disease (4–6, 10, 11). Longitudinal studies have indicated that Tfh cell expansion in blood is most evident early in disease and may decrease over time, while Tph cell expansion persists in established disease (12). In a cohort of patients with lupus nephritis, the frequency of Tph cells, but not Tfh cells, correlated with both disease activity and the frequency of circulating ABCs (4). The developmental relationship between Tph and Tfh cells remains largely unknown.

One challenge in evaluating the relationships between these cell populations is that distinguishing B cell-helper T cells from other activated T cell populations phenotypically remains difficult. Tfh cells express CXCR5, a chemokine receptor that detects the ligand CXCL13 to induce cell migration into secondary lymphoid organ follicles (13–16). More refined populations of active Tfh cells or germinal center Tfh cells are defined by expression of high levels of expression of PD-1, ICOS, BCL6, and CXCR5 (14–17). Tfh cells require the transcription factor BCL6, such that absence of BCL6 prevents accumulation of Tfh cells in vivo (18–20). Identifying Tph cells, which can be functionally defined as B cell-helper T cells that lack CXCR5 and instead express migratory programs targeting non-lymphoid tissues, is more challenging. Tph cells are often quantified in patient samples as CXCR5^−^ PD-1^hi^ CD4 T cells (3–6); however, this gating strategy is likely to capture heterogenous populations that include other activated T cells. In patients with SLE and rheumatoid arthritis (RA), the CXCR5^−^ PD-1^hi^ T cell population is enriched for a B cell-helper transcriptomic signature, and these cells provide help to B cells in vitro, indicating a clear B cell-helper phenotype within this cell population in these diseases (3, 4). However, the proportion of B cell-helper T cells versus other T cell effectors among circulating PD-1^hi^ T cells may vary across diseases. Single cell RNA-seq studies have demonstrated transcriptomically separate populations of Tph and Tfh cells within inflamed tissue (21), both with B cell-helper transcriptional signatures, yet the ability to distinguish these cells transcriptomically in blood has not been established.

In addition to transcriptomic parallels, developmental links between T cell populations in human studies can be inferred from TCR profiling, which provides a tool to track T cell clones across space, time, and phenotype. Each T cell produced in the thymus expresses a unique TCR, which is passed on to all progeny from that T cell clone. The TCRs can thus be utilized as molecular barcodes, enabling the tracking of each T cell’s developmental trajectory. This clonal tracking approach can also be applied to longitudinal samples, making it possible to trace how specific T cell subsets evolve over time and into different subsets. Applied to samples from autoimmune disease patients, this approach has been used to identify shared TCRs in target tissues and blood of patients with type I diabetes and expansion of antigen-specific TCRs during progression to disease (22, 23), as well as the persistence of expanded T cell clones in the circulation of patients with systemic sclerosis over time (24).

Here, we report detailed profiling of TCRs of specific T cell populations from the circulation of patients with SLE, including Tph cells, Tfh cells, T regulatory cells (Tregs), and comparator T cell populations, obtained at diagnosis, 6 months, and 1 year. We combined this with single-cell RNA/TCR profiling for additional cellular resolution to interrogate clonal relationships between Tph and Tfh cell populations in SLE patients. We further profiled Tph and Tfh cells from lung and spleen of mice with pristane-induced lupus to track and manipulate the development of these cells in vivo. These studies demonstrate a strong TCR repertoire overlap between Tph and Tfh cells in SLE patients and in murine pristane-induced lupus across different time points and different tissues, indicating a tight developmental link between Tph and Tfh cells.

## Results

### Persistent shared clones in bulk sorted Tph and Tfh cells from patients with SLE

**Extended Data Figure 1.**
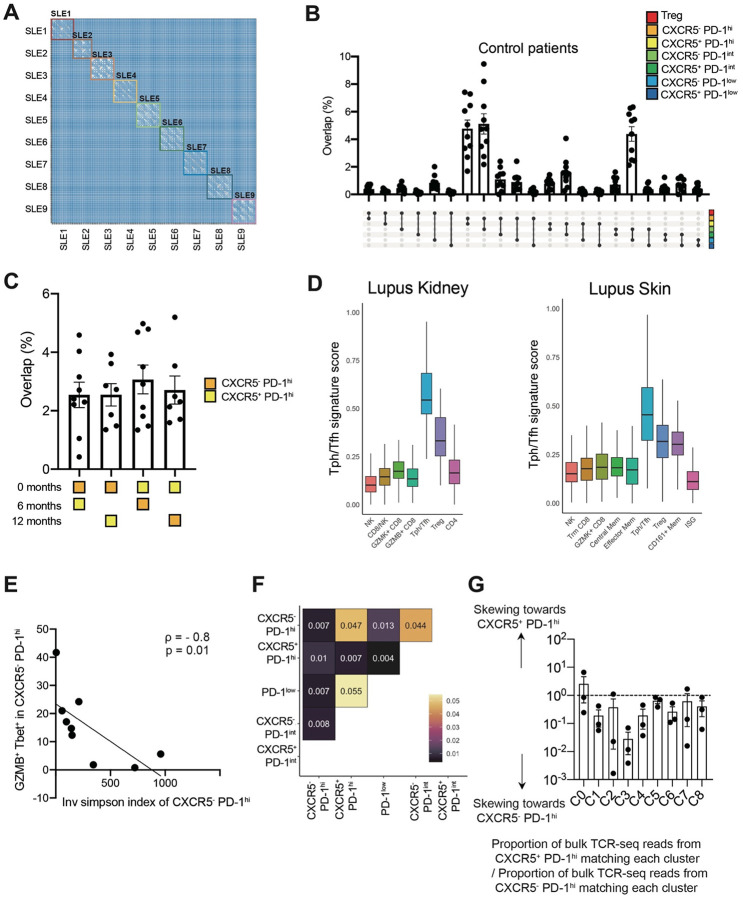
TCR repertoire features in SLE patients **A**. Heatmap of TCR repertoire overlap based on CDR3 beta similarity across all samples from longitudinal SLE patients (n = 378 samples). **B**. TCR repertoire overlap between Tph cells, Tfh cells, Tregs, and 4 other comparator CD4 T cell subsets in non-inflammatory controls (n = 10, downsampled to 5000 reads). **C**. Frequency of unique TCR repertoire to Tph or Tfh cells at baseline detected at 6 months or 1 year (Time B : n = 9, Time C : n = 7, downsampled to 5000 reads). **D**. Tissue Tph/Tfh score of lupus kidney scRNAseq clusters (26) and lupus skin scRNAseq clusters (27). **E**. Correlation analysis between Inversion Simpson Index (shown in Figure 1B) and frequencies of GZMB^+^ Tbet^+^ cells in CXCR5^−^ PD-1^hi^ Tph cells using 9 baseline SLE samples. **F**. TCR repertoire overlap across flow-sorted populations: Tph, Tfh, CXCR5^+^ PD-1^int^, CXCR5^−^ PD-1^int^, and PD-1^low^ cells. **G**. The ratio of the proportion of CXCR5^−^ PD-1^hi^ bulk TCR-seq reads matching scTCRseq in each cluster to the proportion of CXCR5^+^ PD-1^hi^ bulk TCR-seq reads matching scTCRseq in each cluster (n = 3).

**Figure 1.**
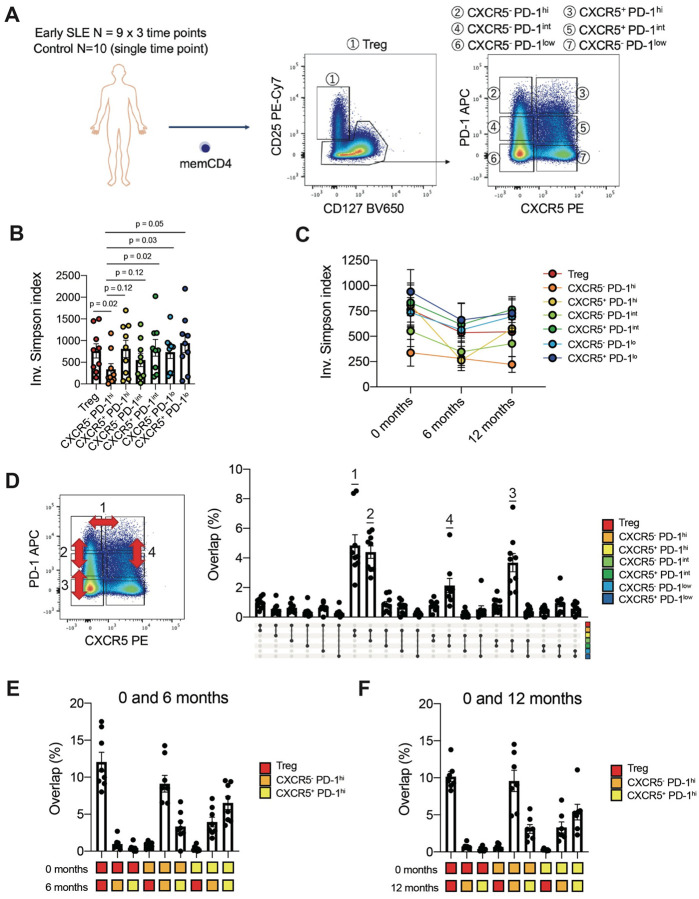
Longitudinal changes of TCR repertoire features in SLE **A**. Schematic of TCR repertoire analysis in SLE. Bulk TCRs of Tph cells, Tfh cells, Tregs, and 4 other comparator CD4 T cell subsets were generated from blood of 9 patients with a new diagnosis of SLE, followed across 3 timepoints and 10 control donors. **B, C**. TCR clonality changes over time (SLE: n = 9). TCR clonality was assessed by calculating the mean of inversion Simpson Index (downsampled to 5000 reads) for the duplicates. P-values by Wilcoxon test. **D**. TCR repertoire overlap between Tph cells, Tfh cells, Tregs, and 4 other comparator CD4 T cell subsets in SLE (n = 9, downsampled to 5000 reads). **E**. TCR repertoire overlap between Tph cells, Tfh cells, and Tregs across baseline and 6 months (SLE: n = 9, downsampled to 5000 reads). **F**. TCR repertoire overlap between Tph cells, Tfh cells, and Tregs across baseline and 1 year (SLE: n = 7, downsampled to 5000 reads).

To compare the TCR repertoires of specific T cell populations and their changes over time in SLE, we performed TCR sequencing on T cell subsets sorted from PBMC of 9 patients with a new diagnosis of SLE. Samples were collected within 6 months of diagnosis, and then 6 months and 12 months later. TCR repertoire analysis was conducted on 7 memory CD4 T cell populations (CD25^+^ CD127^−^ [Treg], CXCR5^−^ PD-1^hi^ [Tph], CXCR5^+^ PD-1^hi^ [Tfh], CXCR5^−^ PD-1^int^, CXCR5^+^ PD-1^int^, CXCR5^−^ PD-1^low^, and CXCR5^+^ PD-1^low^) (Figure 1A). Cells sorted as Tph cells (CXCR5^−^ PD-1^hi^) exhibited the highest degree of clonal expansion, with persistent clonal expansion at each timepoint (Figure 1B, C). Comparison of the overlap of TCR repertoires across different subsets demonstrated substantial clonal overlap across T cell subsets within each patient (Extended Data Figure 1A). We observed significant TCR repertoire overlap between CXCR5^−^ PD-1^hi^ and CXCR5^+^ PD-1^hi^ cells (Figure 1D, indicated as ‘1’), as well as between CXCR5^−^ PD-1^hi^ and CXCR5^−^ PD-1^int^ cells (indicated as ‘2’), between CXCR5^−^ PD-1^int^ cells and CXCR5^−^ PD-1^low^ cells (indicated as ‘3’), and between CXCR5^+^ PD-1^hi^ and CXCR5^+^ PD-1^int^ cells (indicated as ‘4’). The significant repertoire overlap between CXCR5^+^ PD-1^hi^ and CXCR5^−^ PD-1^hi^ cells, which are well separated cytometrically, supports a developmental relationship between Tph and Tfh cells. TCRs of Tregs showed little clonal overlap with any other T cell subset. There was minimal TCR overlap across different patients, as expected (Extended Data Figure 1A). TCR profiling of T cell subsets from a cohort of 10 non-inflammatory control patients demonstrated a similar pattern of TCR repertoire overlap across cell subsets, suggesting that the clonal relationships between T cell populations are not disease-specific (Extended Data Figure 1B).

Longitudinal analyses of T cell subsets from the SLE cohort indicated that CXCR5^−^ PD-1^hi^, CXCR5^+^ PD-1^hi^, and Treg populations have expanded TCR clones that persist over at least the first year after diagnosis (Figure 1E, F). The repertoire overlap between CXCR5^−^ PD-1^hi^ and CXCR5^+^ PD-1^hi^ cells was consistently observed when comparing samples from diagnosis versus 6 months later, and comparing diagnosis versus 1 year later (Figure 1E, F). In addition, unique TCR clones from CXCR5^+^ PD-1^hi^ cells, not shared with CXCR5^−^ PD-1^hi^ cells at baseline, were also detected in CXCR5^−^ PD-1^hi^ cells, and vice versa, at 6 months and 1 year (Extended Data Figure 1C), suggesting potential phenotype transitions within clones. Treg clones remained unique to the Treg subset without substantial appearance in CXCR5^−^ PD-1^hi^, CXCR5^+^ PD-1^hi^, or other T cell subset repertoires over time.

### A cluster of transcriptomically active B cell-helper T cells within Tph and Tfh cell populations

**Extended Data Figure 2.**
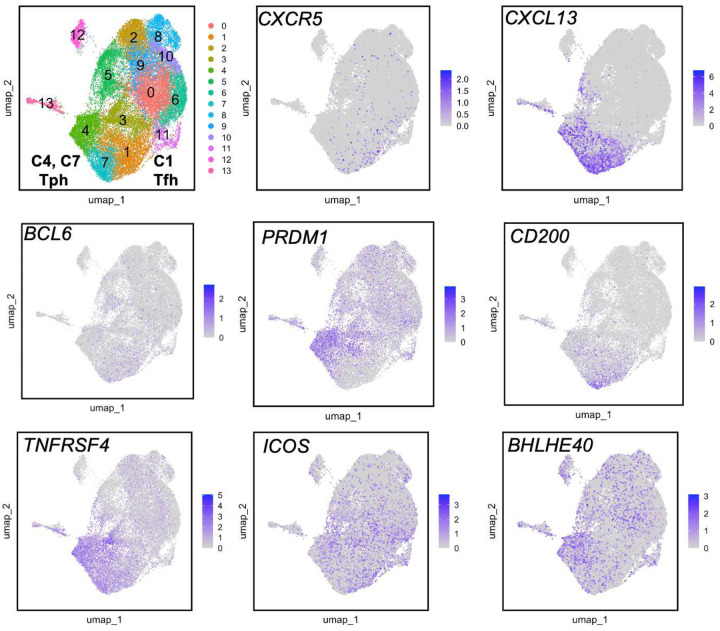
Feature plots of B cell helper genes in CD4 T cells from human RA synovium Expression of several genes associated with Tph (cluster 4 and 7) and Tfh (cluster 1) cells in human CD4 T cells from RA synovium.

**Figure 2.**
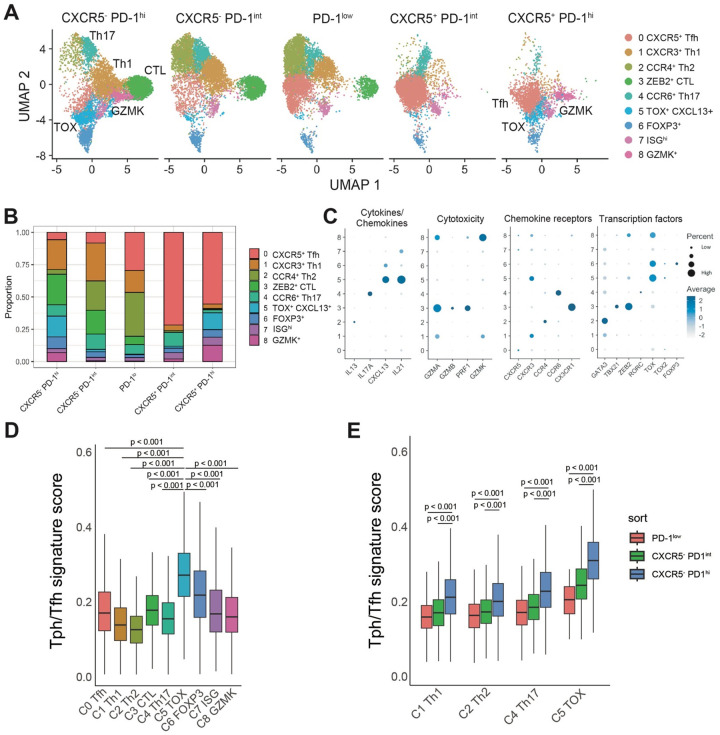
Single cell RNA-seq revealed activated B cell helper T cells **A, B**. Cluster composition of Tph, Tfh, CXCR5^−^ PD-1^int^, CXCR5^+^ PD-1^int^, and PD-1^low^ cells (SLE: n = 3). Tph, Tfh, CXCR5^−^ PD-1^int^, CXCR5^+^ PD-1^int^, and PD-1^low^ cells were sorted, and stained with Hashtag antibodies, and then generated the scRNA-seq data. **C**. Gene expression profiles of each cluster in Lupus PBMC scRNA-seq data. **D**. Tissue Tph/Tfh score of each cluster. **E**. Tissue Tph/Tfh score of Th1, Th2, Th17, and TOX^+^ CXCL13^+^ cells in PD-1^low^, CXCR5^−^ PD-1^int^, and CXCR5^−^ PD-1^hi^ populations.

To evaluate TCRs of cells sorted as Tph and Tfh cells at higher cellular resolution, we conducted scRNA/TCRseq on T cell subsets from 3 of the SLE patients in this cohort using PBMC collected at 18 months after enrollment. We selected 3 patients with a high degree of clonality in PD-1^hi^ cells for analysis. To unambiguously distinguish between CXCR5^−^ PD-1^hi^ (Tph) cells and CXCR5^+^ PD-1^hi^ (Tfh) cells, we sorted CXCR5^−^ PD-1^hi^, CXCR5^+^ PD-1^hi^, CXCR5^−^ PD-1^int^, CXCR5^+^ PD-1^int^, and PD-1^low^ cells from non-Treg memory CD4 T cells, and then generated scRNA/TCRseq data on each sorted population. UMAP visualization of all cells identified 9 clusters (Figure 2A–C): C0 (Tfh), C1 (Th1), C2 (Th2), C3 (cytotoxic T lymphocyte; CTL), C4 (Th17), and C5 (TOX^+^ CXCL13^+^). Over 50% of CXCR5^+^ PD-1^hi^ cells clustered in C0. In addition, 13% of CXCR5^+^ PD-1^hi^ cells clustered in C5 and 12% clustered in C8 (*GZMK*^+^). The C5 cluster expressed several genes associated with B cell-helper function, including *CXCL13*, *IL21*, *CD200*, and *ICOS*. In contrast to Tfh cells, CXCR5^−^ PD-1^hi^ cells were more heterogenous, with cells localized to several different clusters. 16% of these cells localized to C5, such that only cells sorted as PD-1^hi^ Tph or Tfh cells substantially contributed to the C5 cluster representing active B cell-helper T cells. To confirm the identity of C5 cells as activated B cell-helper T cells, we established a tissue Tph/Tfh signature score based on the top 100 genes distinctively expressed by Tph/Tfh clusters from rheumatoid arthritis synovial tissue, where Tph cells were initially described (3, 25) (C1, C4 and C7 in Extended Data Figure 2). This Tph/Tfh signature score well highlighted the tissue Tph/Tfh clusters in kidney (26) and skin (27) in SLE (Extended Data Figure 1D). In addition, the Tph/Tfh signature score was highest in C5, indicating that this cluster expresses a prototype Tph/Tfh signature (Figure 2D). In addition, both PD-1^hi^ populations contributed to the C8 *GZMK*^+^ cluster. In contrast, a separate CTL cluster primarily contained cells from CXCR5^−^ PD-1^hi^ and CXCR5^−^ PD-1^int^ populations, without a contribution from PD-1^hi^ CXCR5^+^ cells. Across each of Th1, Th2, Th17, and TOX^hi^ CXCL13^hi^ clusters, CXCR5^−^ PD-1^hi^ tended to have higher scores for the Tph gene signature as compared to CXCR5^−^ PD-1^int^ or PD-1^low^ cells (Figure 2E).

### TCR clonal overlap between circulating Tph and Tfh cell populations

**Figure 3.**
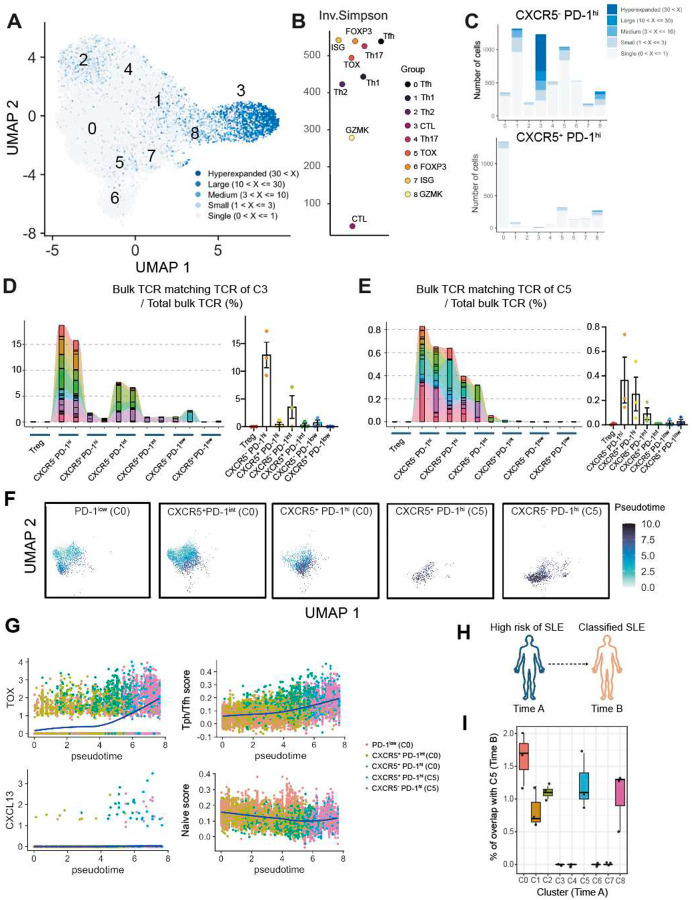
Clonal and developmental link between Tfh and Tph cells **A**. Clonality of each cluster in Lupus PBMC scRNAseq data. Cells were classified based on the number of TCR clones detected. **B**. Inverse Simpson Index of each cluster. **C**. Clonality of each cluster divided by sorted Tph and sorted Tfh cells. **D**. Frequencies of TCR repertoire of C3 from patient SYL3 detected in bulk TCR-seq data at enrollment. Technical duplicate data are shown. **E**. Frequencies of TCR repertoire of C5 from SYL3 detected in bulk TCR-seq data at enrollment. **F**. Pseudotime analysis between C0 and C5 divided in to PD-1^low^, CXCR5^−^ PD-1^int^, sorted Tfh and sorted Tph cells. **G**. Trend of gene expression, Tph/Tfh signature score, naive gene signature scores aligned along pseudotime. **H**. Experirmental scheme of the pre-SLE cohort. **I**. TCR overlap frequency between each subset at time A (pre-onset) and C5 at time B (post-onset).

We then assessed the TCR clonal relationships across the finer T cell clusters revealed by scRNA-seq. C3 containing CTL exhibited the most clonal expansion (Figure 3A, B). The presence of these highly clonally expanded CTLs accounts for the high oligoclonality of CXCR5^−^ PD-1^hi^ cells observed in Figure 1. Consistent with this, the extent of clonality of bulk CXCR5^−^ PD-1^hi^ pool was strongly correlated with the frequency of Tbet^+^ granzyme B^+^ CXCR5^−^ PD-1^hi^ CD4 T cells as quantified by mass cytometry analysis of the same samples (Extended Data Figure 1E). We then assessed the TCR clonality of CXCR5^−^ PD-1^hi^ or CXCR5^+^ PD-1^hi^ cells within each scRNA-seq cluster. C3 (CTL) from CXCR5^−^ PD-1^hi^ cells exhibited the most oligoclonality, while large expanded clones could also be found in C5 (*TOX*^+^ *CXCL13*^+^), as well as C1 (Th1) and C8 (*GZMK*^+^) (Figure 3C).

**Extended Data Figure 3.**
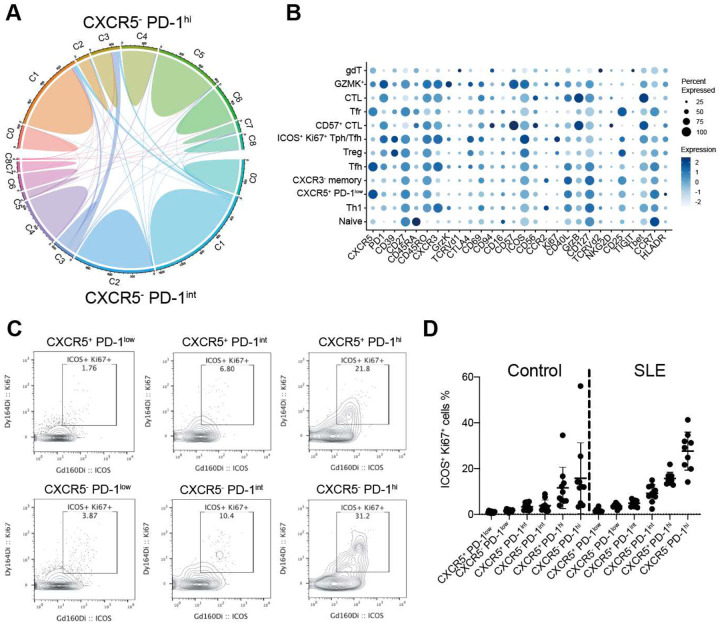
TCR overlap and mass cytometric validation of C5 detected by scRNAseq **A**. Circos plot showing the TCR sequence overlap in each cluster between CXCR5^−^ PD-1^hi^ cells and CXCR5^−^ PD-1^int^ cells. **B**. Marker expression of each cluster in the UMAP. **C**. ICOS^+^ Ki67^+^ cells in CXCR5^−^ PD-1^hi^ Tph, CXCR5^+^ PD-1^hi^ Tfh, CXCR5^−^ PD-1^int^, CXCR5^+^ PD-1^int^, CXCR5^−^ PD-1^low^, CXCR5^+^ PD-1^low^ cells. **D**. Frequencies of **C** in 10 non-inflammatory controls and baseline 9 SLE samples.

Analysis of the overall clonality of sorted populations using the scRNA-seq data recapitulated the same patterns of clonal overlap as observed in the bulk TCR analyses, including repertoire overlap between CXCR5^−^ PD-1^hi^ and CXCR5^+^ PD-1^hi^ cells and between PD-1^low/int/hi^ CXCR5^−^ populations (Extended Data Figure 1F). To determine the specific cell clusters that account for the clonal overlap between CXCR5^−^ PD-1^hi^ and CXCR5^+^ PD-1^hi^ cells observed in the bulk TCR data, we used the scTCR data to determine the proportion of TCRs in each single cell cluster that was detected in bulk TCR-seq data from the enrollment time point. TCRs from cluster C3 (CTL) were predominantly associated with the bulk CXCR5^−^ PD-1^hi^ pool and were not found in the bulk CXCR5^+^ PD-1^hi^ pool (Figure 3D). In contrast, TCRs from C5 (TOX^+^ CXCL13^+^) were found in both bulk CXCR5^−^ PD-1^hi^ and CXCR5^+^ PD-1^hi^ pools (Figure 3E). This pattern of shared Tph/Tfh clones in C5 but not in C3 was consistent across the 3 SLE patients (Figure 3D, E, Extended Data Figure 1G), indicating that cells with a B cell-helper signature, and not CTL, account for the clonal overlap in TCRs of bulk CXCR5^−^ PD-1^hi^ and CXCR5^+^ PD-1^hi^ pools. In addition, TCRs from C1 (Th1) and C3 (CTL) were shared between the CXCR5^−^ PD-1^hi^ pool and the CXCR5^−^ PD-1^int^ pool (Extended Data Figure 3A). In total, these results indicate shared B cell-helper T cell clones between PD-1^hi^ Tph and Tfh cells, distinguishable from large CTL clones found exclusively in the CXCR5^−^ PD-1^hi^ pool and not in CXCR5^+^ PD-1^hi^ Tfh cells.

To interrogate the direction of differentiation between C0 and C5, we performed a pseudotime analysis. The pseudotime visualization indicated a trajectory from PD-1^low^ cells in C0 to CXCR5^+^ PD-1^int^ in C0, CXCR5^+^ PD-1^+^ in C0, CXCR5^+^ PD-1^+^ in C5, then CXCR5^−^ PD-1^+^ in C5, with gradually increased expression of *TOX*, *CXCL13*, and the tissue Tph/Tfh signature, but a decreased naive T cell signature along this trajectory (Figure 3F, G). Taken together, these TCR overlap results and pseudotime analysis suggest a potential transition from Tfh to Tph cell phenotypes.

### Tph/Tfh cell clones are expanded prior to full disease onset

**Extended Data Figure 4.**
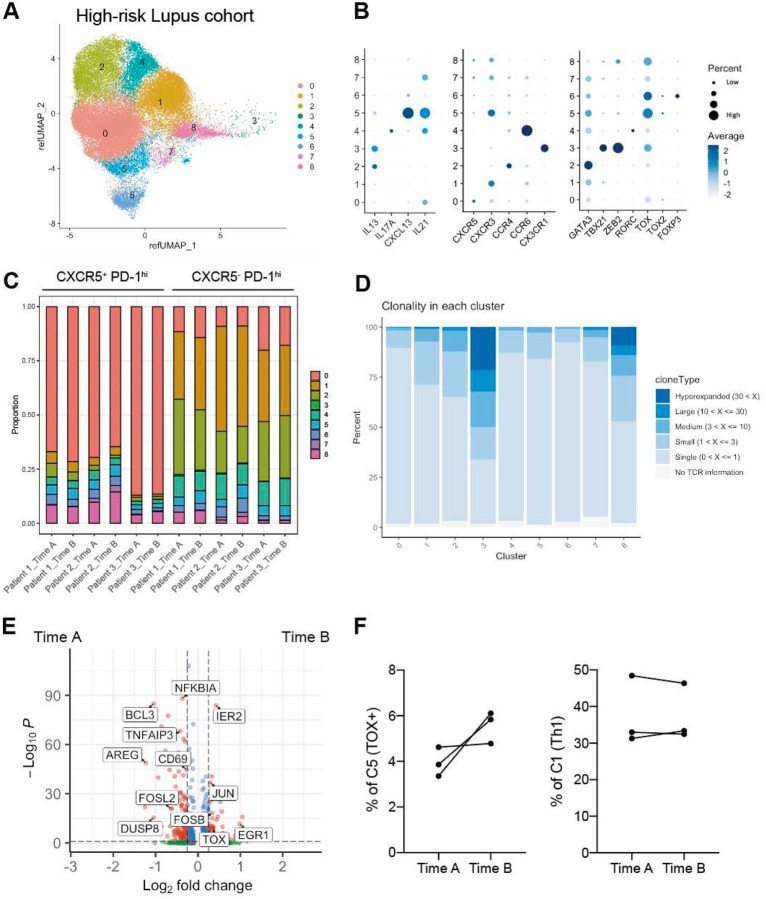
scRNAseq analysis of the high risk Lupus cohort **A**. UMAP of CXCR5^−^ PD-1^hi^ cells and CXCR5^+^ PD-1^hi^ cells from the high-risk for lupus cohort. **B**. Gene expression profiles of each cluster in the high-risk for lupus scRNA-seq data. **C**. Cluster abundances in CXCR5^−^ PD-1^hi^ cells and CXCR5^+^ PD-1^hi^ cells from each sample. **D**. Clonality of each cluster in high-risk Lupus PBMC scRNAseq data**. E**. Volcano plot for the DEGs of CXCR5^−^ PD-1^hi^ cells between before (Time A) and after (Time B) the classification of SLE. **F**. Frequencies of C5 and C1 before (Time A) and after (Time B) meeting classification criteria for SLE.

To evaluate whether clonal expansion of Tph and Tfh cells occurs prior to the full clinical manifestation of SLE, we evaluated CD4 T cells longitudinally from a set of patients who did not initially meet the ACR/EULAR 2019 classification criteria for SLE, but scored 4–9 points, and were considered clinically at high risk of developing SLE. From this cohort, we identified 3 individuals who provided blood samples before (‘high risk of SLE’) and after meeting classification criteria for SLE (‘classified SLE’). We sorted CXCR5−PD-1^hi^ and CXCR5+PD-1^hi^ cells to generate scRNA/TCR-seq and projected the scRNA-seq data onto the reference SLE UMAP (Extended Data Figure 4A, B). Consistent with the SLE data (Figure 2A), the CXCR5^+^ PD-1^hi^ population from these patients was predominantly composed of C0 (Tfh), whereas sorted CXCR5^−^ PD-1^hi^ cells contained cells that clustered in C1 (Th1), C2 (Th2), and C4 (Th17). Again, the B cell helper population C5 was present in both sorted CXCR5^+^ and CXCR5^−^ PD-1^hi^ cell populations (Extended Data Figure 4C). TCR clonality analysis confirmed that the most clonally expanded populations were C3 (CTL) and C8 (GZMK) (Extended Data Figure 4D), though the proportion of CXCR5^−^ PD-1^hi^ cells that clustered in C3 was smaller in this dataset.

To investigate transcriptional changes associated with SLE onset, we performed differential gene expression analysis of CXCR5−PD-1^hi^ from the pre- and post-classified SLE timepoints. Post-SLE classification cells showed upregulation of TOX and downregulation of NFKBIA and TNFAIP3, negative regulators of NF-κB signaling, as well as CD69, an early activation marker (Extended Data Figure 4E). Consistent with these changes, cluster abundance analysis revealed a marked increase in the C5 (*TOX*^+^ CXCL13^+^) population within the CXCR5^−^ PD-1^hi^ subset in all 3 patients after disease onset (Extended Data Figure 4F).

We then used the scTCR-seq data to determine whether clones expanded at the classified SLE timepoint were also present at the high-risk (pre-classified SLE) timepoint. Of 6310 expanded clones in sorted CXCR5^−^ PD-1^hi^ cells post-SLE classification, 60.8% were also detected at the high-risk, pre-classified timepoint. Similarly, of 2447 expanded clones in sorted CXCR5^+^ PD-1^hi^ cells post-disease classification, 54.6% were also detected at the high-risk, pre-classified timepoint. This indicates that expanded Tph and Tfh cell clones are present even before full manifestation of SLE disease. To further explore the potential origins of the clones in C5, we calculated the overlap of TCR sequences between post-onset C5 and each pre-onset cluster. This analysis demonstrated that C0 exhibited the highest degree of overlap, followed by C5 and C8 (Figure 3I), suggesting that C0 may provide one pool for emergence of the C5 population concurrent with disease onset.

### TOX^+^ IL21^+^ CXCL13^+^ T cells represent an activated proliferative B cell helper population in SLE

**Figure 4.**
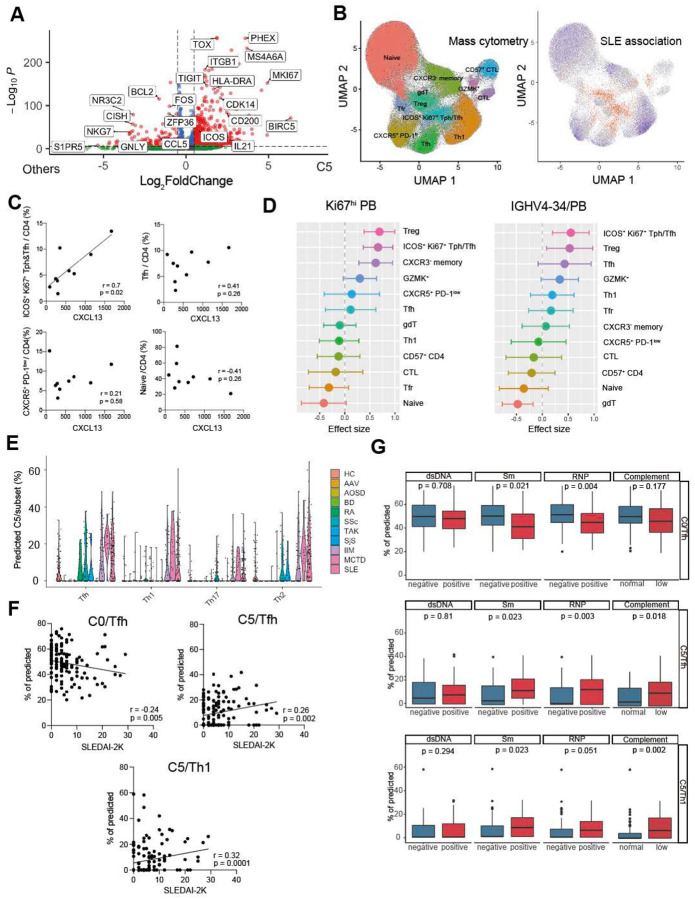
C5 is a strong B cell activator population **A**. Volcano plot for the DEGs between C5 and other clusters. Red: adj p values < 0.05, log2FC > 0.5, Green: adj p values > 0.05, Blue: adj p values < 0.05, log2FC < 0.5. **B. Left:** UMAP CD4 T cell clustering of mass cytometry from 14 non-inflammatory controls and 9 SLE patient samples. **Right:** CNA of mass cytometry data corrected for age and sex. N =14 non-inflammatory controls, 9 SLE patients (baseline samples). Red indicates neighborhoods positively associated with SLE, and blue indicates negative association with SLE. **C**. Correlation between serum CXCL13 and the frequencies of ICOS^+^ Ki67^+^ Tph/Tfh cluster, Tfh cluster CXCR5^+^ PD-1^low^ cluster, and naive cluster at the baseline SLE samples. **D**. Linear mixed effect model analysis between mass cytometry clusters and the frequencies of IGHV4–34 on PB quantified by BCR-seq and Ki67^+^ PB quantified by mass cytometry. 27 SLE patient samples (9 SLE patients x 3 time points) were used, with time as fixed effect. **E**. Estimated proportion of C5 cells among total cells by deconvolution of ImmuNexUT bulk RNAseq from 495 donors (HC, healthy controls: 79, AAV, ANCA-associated vasculitis:22, AOSD, adult-onset Still’s disease: 17, BD, Behcet’s disease : 22, IIM, idiopathic myositis: 64, RA, rheumatoid arthritis:24, SSc, systemic sclerosis:64, TAK, Takayasu disease:16, MCTD, mixed connect tissue disease: 19, SjS, Sjogren syndrome:18, SLE, systemic lupus erytomatosus:136). **F**. Spearman’s correlation between the estimated frequencies of C0 and C5 in ImmuNexUT study and SLEDAI-2K (n = 136). **G**. Frequencies of C0 and C5 in the SLE patients with or without autoantibodies and hypocomplementemia. Student t-test was used for the comparisons.

scRNA-seq analyses highlighted a population of T cells, captured in cluster C5, that expressed multiple B cell-helper features and was found specifically among CXCR5^−^ PD-1^hi^ Tph and CXCR5^+^ PD-1^hi^ Tfh cells. We surmised that the scRNA-seq analysis revealed a population of functionally active B cell-helper T cells in the circulation that is otherwise difficult to visualize; therefore, we sought to identify specific markers to more precisely capture the C5 population by cytometry. Analysis of differentially expressed genes (DEG) revealed *TOX*, *CD200*, *ICOS*, and proliferation markers such as *MKI67* and *CDK14* as preferentially expressed in C5 (Figure 4A), consistent with an activated, proliferative B cell-helper population. We then evaluated expression patterns of these markers in a mass cytometry dataset generated from the same patient cohort (12). ICOS^+^ Ki67^+^ cells were found to be most abundant in gated CXCR5^−^ PD-1^hi^ cells and least abundant in PD-1^low^ cells, consistent with the scRNA-seq data (Extended Data Figure 3B, C, Figure 2A). Unsupervised clustering of cells in the mass cytometry dataset also identified a population of ICOS^+^ Ki67^+^ Tph/Tfh cells (Figure 4B, Extended Data Figure 3B). To test their association with SLE, we applied a Covarying Neighborhood Analysis (CNA) (28), which demonstrated that ICOS^+^ Ki67^+^ Tph/Tfh cells, Tregs, and granzyme K^+^ CD4 T cells were significantly enriched in SLE patients compared to controls (Figure 4B, Extended Data Figure 3D). The proportion of ICOS^+^ Ki67^+^ Tph/Tfh cells was also significantly associated with the serum CXCL13 level, whereas that of total CXCR5^+^ PD-1^hi/int^ Tfh cells was not (Figure 4C). ICOS^+^ Ki67^+^ Tph/Tfh cells were also significantly associated with the frequency of Ki67^+^ plasmablasts quantified by mass cytometry and IGHV4–34, a well-known autoreactive BCR-IGHV chain (29, 30), quantified by bulk BCR-seq (Figure 4D). Together, these results indicate that tracking an ICOS^+^ Ki67^+^ Tph/Tfh population captures an activated T cell population linked to overall T-B cell activation in SLE.

Next, to evaluate the C5 signature in additional cohorts of patients with SLE and other autoimmune diseases, we performed a deconvolution analysis using publicly available bulk RNA-seq data of CXCR3^+^ Th1, CCR4^+^ Th2, CCR6^+^ Th17, and CXCR5^+^ Tfh cells from 495 donors, including healthy controls and patients with 337 autoimmune diseases (31, 32). Tph cells are not available in this dataset. C5 cells identified by deconvolution were observed in Th1, Th2, Th17, and Tfh populations, with a highest representation in the Tfh cell population. Compared to healthy controls, the proportion of C5 cells was increased in SLE, mixed connective tissue disease, and idiopathic inflammatory myositis patient samples (Figure 4E). To further evaluate the association between the C5 population and plasmablasts, we examined the correlation using bulk RNA-seq data of plasmablasts from 136 SLE patients in this dataset (31, 32). Additionally, the frequency of C5 in Tfh cells and non-Tfh cells (Th1) showed a positive correlation with SLEDAI, whereas the frequency of C0 cells in Tfh cells was negatively correlated (Figure 4F). This inverse relationship between C0 and C5 suggests a potential differentiation shift from C0 to C5 in patients with higher disease activity. Furthermore, C5 frequency tended to be higher in patients who were positive for autoantibodies such as anti-RNP antibody and anti-Sm antibody, as well as in those with hypocomplementemia, supporting the role of C5 in promoting autoantibody production (Figure 4G).

### CXCR5^−^ PD-1^hi^ B cell-helper T cells accumulate in murine lupus

**Figure 5.**
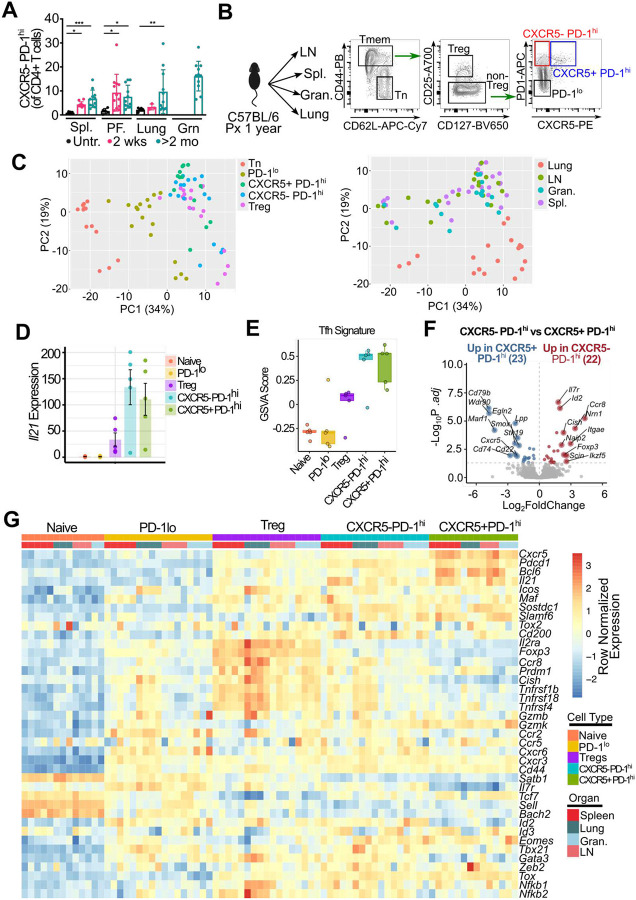
CXCR5^−^ PD-1^hi^ CD4 T cells in mouse have a B cell helper signature **A**. Quantification of CXCR5^−^ PD-1^hi^ subset in pristane induced lupus in spleen (Spl), peritoneal fluid (PF), lung and granuloma (Grn) at indicated time-points. ANOVA results with indicated post-test significance. * p = <0.05, ** p = < 0.01, *** p = <0.001, n= 3–4 per experiment. Pool of 3 independent experiments. **B**. Sorting schematic of the RNA-seq experiment. **C**. PCA analysis of sorted cell subsets grouped by cell type (left) or tissue (right). **D**. Splenic *Il21* expression of normalised counts across each of the sorted subsets. **E**. GSVA analysis using a Tfh gene signature across each of the different CD4 T cell subsets. **F**. DEG analysis of splenic CXCR5^−^ PD-1^hi^ vs CXCR5^+^ PD-1^hi^ subset. Some of the top differentially expressed genes are highlighted. **G**. Heatmap of gene expression for a custom gene list comprised of lineage, functional, migratory and activation markers. Normalised gene expression is plotted (row normalised.

To evaluate whether similar heterogeneity and developmental links are observed among Tph cells in tissues, we evaluated PD-1^hi^ CD4 T cells in the murine model of pristane-induced lupus in C57BL/6 mice, a flexible model of lupus that involves activation of extrafollicular T-B cell interactions and can be induced in multiple strains (33). By two weeks after pristane treatment, a population of cells expressing high levels of PD-1 but lacking CXCR5, resembling human Tph cells, accumulated in spleen and peritoneal fluid (Figure 5A). At time points after two months, these cells became detectable in the lungs, peritoneal fluid, and lipogranulomas within the peritoneal cavity. This population was also present in the spleen, along with Tfh cells.

**Extended Data Figure 5.**
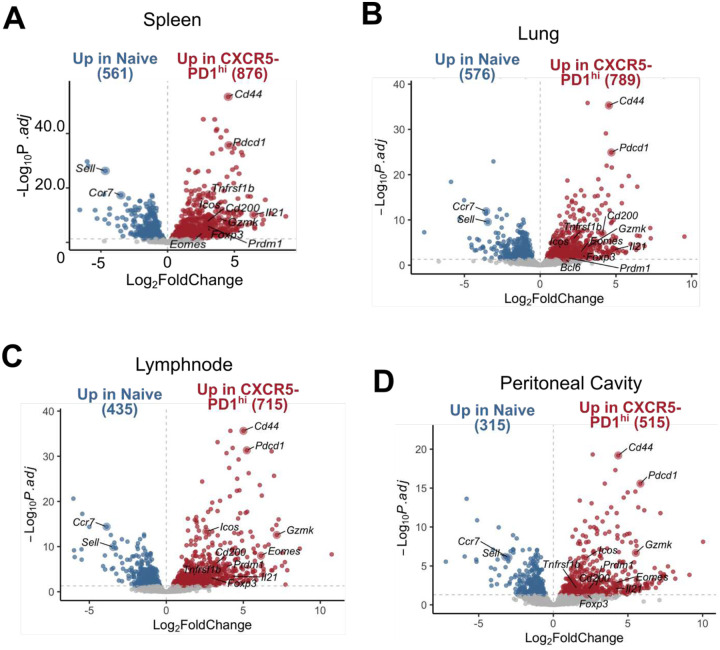
Differential gene expression analysis between CXCR5- PD-1^hi^ and naïve T cells across different tissues **A**. Volcano plot of differentially expressed genes between spleen naïve CD4 T cells and lung CXCR5- PD-1^hi^ CD4 T cells. Several significant DEGs are annotated relating to naïve, Th1, Treg and B cell helper function. **B**. Volcano plot of differentially expressed genes between lung naïve CD4 T cells and lung CXCR5- PD-1^hi^ CD4 T cells. Several significant DEGs are annotated relating to naïve, Th1, Treg and B cell helper function. **C**. Volcano plot of differentially expressed genes between lymph node naïve CD4 T cells and lymph node CXCR5- PD-1^hi^ CD4 T cells. Several significant DEGs are annotated relating to naïve, Th1, Treg and B cell helper function. **D**. Volcano plot of differentially expressed genes between granuloma naïve CD4 T cells and granuloma CXCR5- PD-1^hi^ CD4 T cells. Several significant DEGs are annotated relating to naïve, Th1, Treg and B cell helper function.

To investigate the phenotype of these cells, we sorted T cell subsets from the spleen, draining lymph nodes, peritoneal granulomas, and lungs and performed RNA-seq (Figure 5B). Principal component analysis (PCA) revealed distinct clustering of cell types, with naïve cells, PD-1^low^ cells, and CXCR5^−^ PD-1^hi^/CXCR5^+^ PD-1^hi^/Treg cells forming separate clusters (Figure 5C). This clustering pattern was consistent across all tissues, although cells originating from the lungs clustered separately from other tissues. DEG analysis comparing CXCR5^−^ PD-1^hi^ cells from spleen with naïve T cells revealed upregulation of genes associated with B cell helper function, including *Pdcd1*, *Icos*, and *Il21* in CXCR5^−^ PD-1^hi^ cells (Extended Data Figure 5A) with similar patterns observed in other tissues (Extended Data Figure 5B–C). Expression of *Il21* was equivalently elevated in the CXCR5-PD-1hi and CXCR5+PD-1hi T cell subsets, consistent with a shared B cell helper function (Figure 5D). GSVA analysis using a Tfh signature derived from known positive regulators of Tfh development (34) also showed equal enrichment between CXCR5-PD-1hi and CXCR5+PD-1hi T cell subsets. Finally, comparison of CXCR5^−^ PD-1^hi^ with CXCR5^+^ PD-1^hi^ cells revealed few DEGs, indicating a phenotypic similarity between the two populations.

**Extended Data Figure 6.**
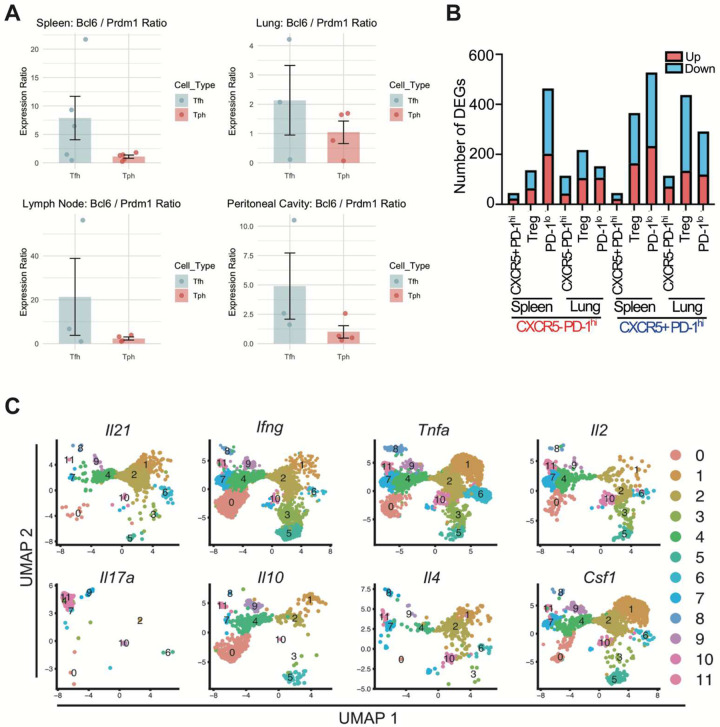
Transcriptional profiles associated with Tfh and Tph cells in spleen and lung **A**. Ratio of Bcl6 / Prdm1 in Tfh and Tph cells in spleen and lung. Normalized counts are shown as mean ± SD for 3–5 mice per group. **B**. Frequency of differentially expressed genes (DEGs) for each pairwise comparison across spleen and lung. Genes upregulated in CXCR5+ PD-1^hi^ (Tfh) cells are shown in red, and genes upregulated in CXCR5− PD-1^hi^ (Tph) cells are shown in blue. **C**. Cytokine expression across CD4 T cell clusters in spleen and lung, visualized by Feature Plots.

Consistent with human Tph cells, murine CXCR5^−^ PD-1^hi^ cells exhibited reduced ratio of *Bcl6* to *Prdm1* (Extended Data Figure 6A). However, within the DEGs (splenic CXCR5^−^ PD-1^hi^ vs naïve T cells), we also observed genes not typically associated with B cell-helper function, such as *Foxp3*, *Gzmk*, and *Eomes*, suggesting heterogeneity within the CXCR5^−^ PD-1^hi^ population (Figure 5D). To explore how similar CXCR5^−^ PD-1^hi^ cells were compared to other populations, we performed differential gene expression analysis against Treg and PD-1^lo^ populations in both the spleen and lung. We observed fewer DEGs when CXCR5^−^ PD-1^hi^ cells were used as the comparator population in contrast to CXCR5^+^ PD-1^hi^ cells (Extended Data Figure 6B). These data suggested that CXCR5^−^ PD-1^hi^ cells shared more transcriptional similarity to Tregs and PD-1^lo^ cells than do CXCR5^+^ PD-1^hi^ cells. Heatmap analysis of z-scored gene expression across CD4^+^ T cell subsets revealed that CXCR5-PD-1hi cells shared transcriptional features with CXCR5^+^ PD-1^hi^ cells, characterized by high expression of *Cxcr5*, *Pdcd1*, *Il21*, and *Icos* (Figure 5G). However, CXCR5^−^ PD-1^hi^ cells also expressed genes typically associated with other subsets, including *Prdm1*, *Gzmb*, and members of the *Tnfrsf*, suggesting phenotypic heterogeneity within this population. This mixed profile was consistent across tissues, although the relative expression of chemokine receptors and effector-associated genes varied by organ. While CXCR5^−^ PD-1^hi^ cells clearly expressed genes important for B cell-helper function, they also expressed genes linked to Tregs, cytotoxicity, and other lineages, suggesting substantial heterogeneity.

### Heterogeneity of B cell-helper T cells in spleen and lung

**Extended Data Figure 7.**
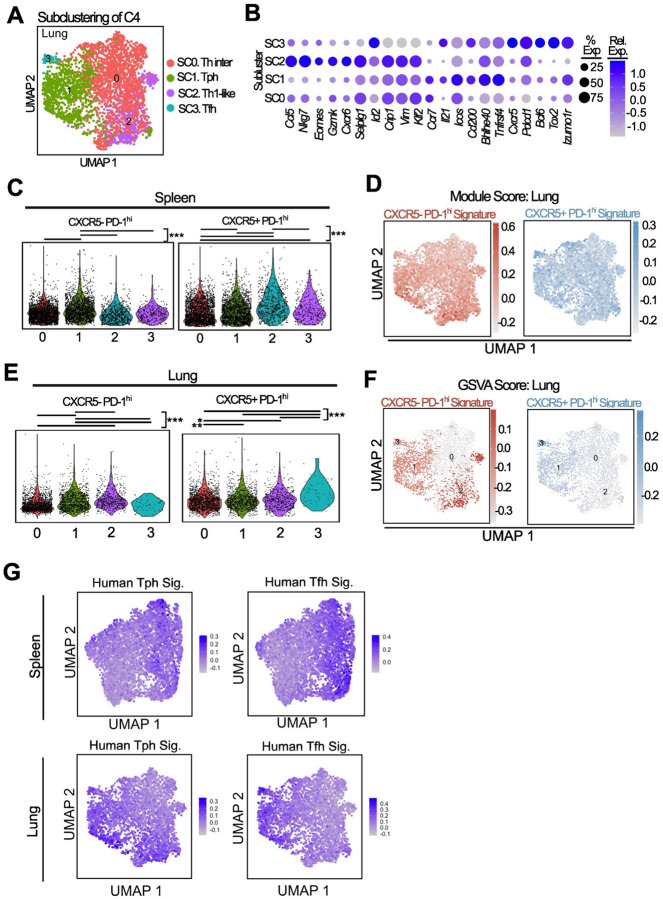
Subcluster analysis reveals distinct transcriptional states within spleen and lung **A**. DimPlot showing subcluster analysis of lung cluster 4. **B**. DotPlot of gene expression for various functional T cell states. **C**. Single cell GSVA scoring of spleen subclusters with ANOVA analysis and Tukey’s post-hoc testing. *** p = <0.001 **D**. UMAP projection showing module scoring of lung cluster 4 subclusters using genes upregulated in CXCR5- PD1^hi^ cells (red) or CXCR5+ PD1^hi^ (blue) derived bulk-sorted populations. **E**. Single cell GSVA scoring of lung subclusters with ANOVA analysis and Tukey’s post-hoc testing.* p = < 0.05, ** p = < 0.01, *** p = <0.001. **F**. UMAP plot of aggregate lung subcluster expression analysed by GSVA for signatures in (Figure 6F). **G**. Module scoring of spleen and lung subclusters using human derived Tph and Tfh signatures.

**Figure 6.**
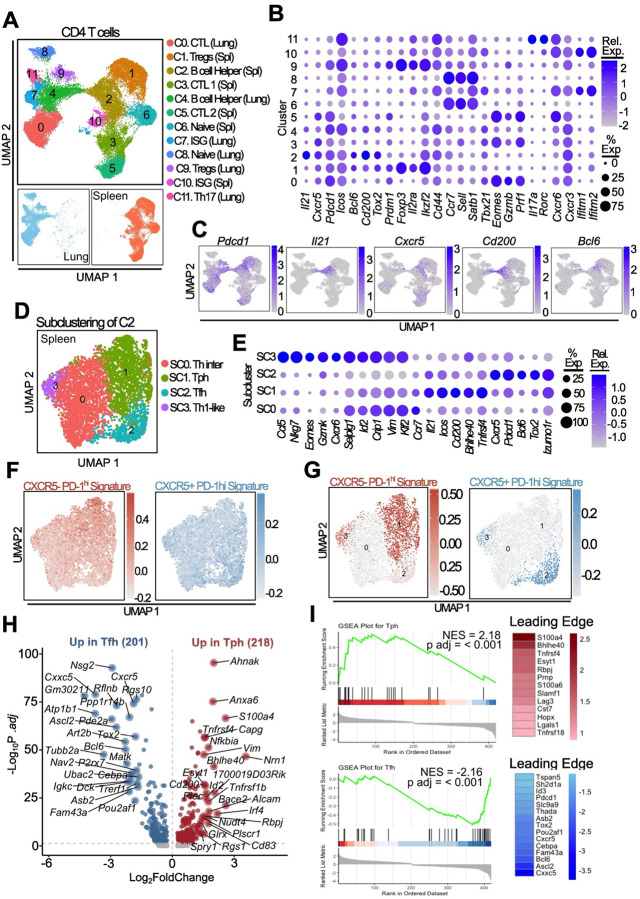
scRNA-seq analysis of CD4 T cells in murine lupus shows B cell helper T cell cluster heterogeneity **A**. UMAP plot showing clustering of cell populations for both spleen and lung tissues. **B**. Dot plot of relative gene expression for various functional T cell states. **C**. Feature plots of single gene expression for labelled genes. **D, E**. UMAP and dot plot of gene expression for spleen cluster 2 sub-clustering. **F**. UMAP projection showing module scoring of splenic cluster 2 subclusters using genes upregulated in CXCR5^−^ PD-1^hi^ cells (red) or CXCR5^+^ PD-1^hi^ (blue) derived bulk-sorted populations. **G**. UMAP plot of aggregate subcluster expression analysed by GSVA for signatures in (F). **H**. Differential gene expression between splenic Tph (subcluster 1) and Tfh (subcluster 2) subsets. Top 25 in each direction are annotated. **I**. GSEA analysis of DEGs in (H) using human derived Tph or Tfh signatures. Shown on the RHS are the genes contributing to the leading edge of the enrichment.

To better resolve the B cell-helper phenotype in CXCR5^+^ PD-1^hi^ and CXCR5^−^ PD-1^hi^ CD4 T cell populations in this model, we analyzed total CD4 T cells from spleen and lung of mice 12 months after pristane treatment by scRNA-seq. UMAP visualization of T cells from both spleen and lung identified 11 clusters (Figure 6A). One cluster in spleen (cluster 2) and one cluster in lung (cluster 4) exhibited high expression of genes associated with B cell-helper function, including *Il21*, *Cxcr5*, *Pdcd1*, *Bcl6* and *Cd200* (Figure 6B). Expression of *Il21* was relatively selective to clusters 2 and 4 (Extended Data Figure 6C). However, expression of these genes varied across the cluster, indicating within-cluster heterogeneity (Figure 6C); therefore, we evaluated each cluster separately to unmask potential subcluster subset diversity. Cells within spleen cluster 2 segregated into four subpopulations (Figure 6D). Subcluster 2 showed a clear Tfh phenotype, with the highest expression of *Bcl6*, *Tox2*, and *Cxcr5* (Figure 6E). Analysis of lung cluster 4 showed a similar pattern, albeit with less distinction between Tph and transition Th inter subsets (Extended Data Figure 7A–B). Spleen subcluster 1 had the highest expression of *Il21*, *Icos*, *Cd200*, *Tnfrs4* and transcription factor *Bhlhe40*, demonstrating some features shared with human Tph cells. Subcluster 0 showed an intermediate differentiation phenotype sharing some B cell helper molecule expression but also overlapping features with a Th1/GzmK state (subcluster 3).

To evaluate the extent to which distinctions between subclusters 1 and 2 paralleled differences between bulk PD-1^hi^ CXCR5^−^ and PD-1^hi^ CXCR5^+^ cells, we evaluated expression of gene signatures defined from the bulk CXCR5^−^ PD-1^hi^ and CXCR5^+^ PD-1^hi^ pools in the single cell data. Genes overexpressed in CXCR5^−^ PD-1^hi^ cells were highly enriched in subcluster 1 (Figure 6F, Extended Data Figure 7C). In contrast, genes overexpressed in the CXCR5^+^ PD-1^hi^ pool showed higher expression in subcluster 2, which most resembled Tfh cells. Analysis of lung subclusters showed a similar pattern to that observed in the spleen (Extended Data Figure 7D–F). Pseudo-bulk GSVA analysis further showed that spleen subcluster 1 enriched for a CXCR5^−^ PD-1^hi^ signature but not a CXCR5^+^ PD-1^hi^ signature, consistent with these cells being distinct from Tfh cells (Figure 6G). Lung Tph cells showed a broader enrichment of the CXCR5^−^ PD-1^hi^ gene signature which included the small number of Tfh cells detected in the lung (Extended Data Figure 7F). Finally, we used human-derived Tph and Tfh signatures to score B cell helper cluster in the spleen and lung. This showed enrichment of a human Tph signature in subcluster 1 of spleen and lung, consistent with these cells being transcriptionally similar with Tph cells (Extended Data Figure 7G).

DEG analysis directly comparing splenic subclusters 1 (Tph) and 2 (Tfh) showed upregulation of Tfh-associated genes *Cxcr5*, *Tox2*, *Bcl6*, *Pou2af1* and *Ascl2* in subcluster 2 (Figure 6H). In contrast, subcluster 1 showed upregulation of genes important for calcium flux and IL-2 signalling (*Ahnak* and *Anxa6* respectively), as well as transcription factors *Id2* and *Bhlhe40*, both negative regulators of *Bcl6*. These cells also showed higher expression of OX40 (*Tnfrsf4*) and TNFR2 (*Tnfrsf1b*). GSEA analysis of a human Tph signature showed significant enrichment of genes upregulated in mouse Tph cells; in contrast murine Tfh cells showed significant enrichment of a tonsil derived Tfh signature (Figure 6I). Together, these results indicate that *Il21*-expressing T cell populations in the spleen and lung contain transcriptomically separable Tph and Tfh subsets.

### Tph and Tfh cells in spleen and lung are clonally linked

**Figure 7.**
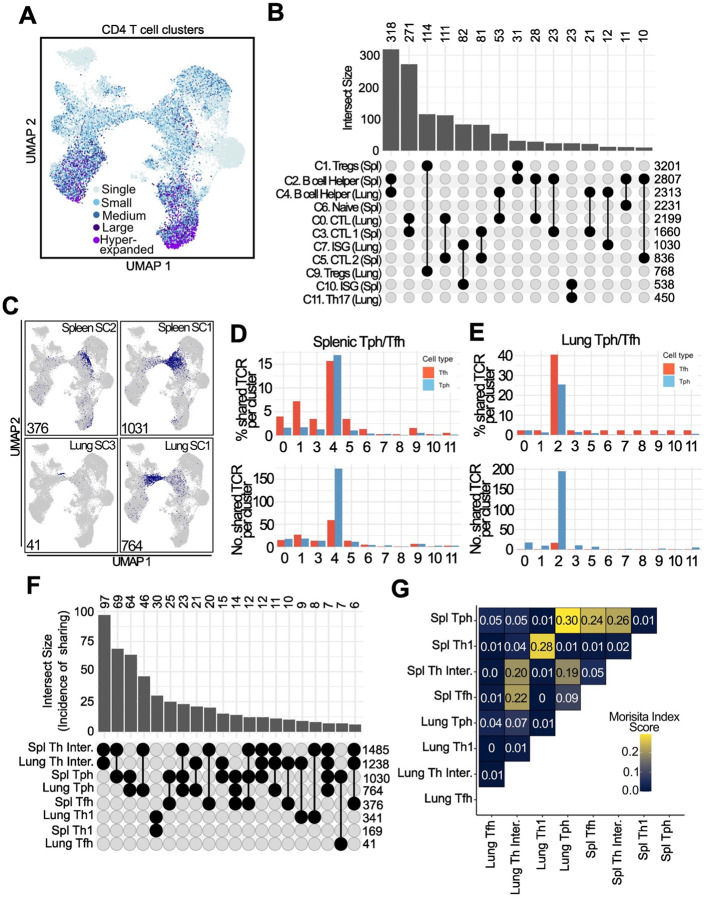
TCR analysis of B cell helper T cells shows high degree of sharing between Tph and Tfh cells in both spleen and lung **A**. TCR expansion across the dataset in both spleen and lung. Single (1), Small (>1–5), Medium (>5–20), large (>20–100) and hyperexpanded (>100) are annotated on the plot. **B**. Upset plot of TCR sharing showing the occurrence of a TCR clones being shared between different clusters (number above bar). Only the top 15 comparisons are shown. Total TCRs for each cluster are shown on the right. **C**. TCR clones from spleen and lung Tph/Tfh subsets mapped onto the UMAP to show sharing across clusters. **D, E**. Quantification of C in both the spleen and lung, showing both the percentage of unique TCRs of either Tph/Tfh cells shared per cluster and the number of unique shared TCRs per cluster. **F**. Upset plot as in B but showing unique TCR sharing between spleen and lung B cell helper subclusters. **G**. Morisita index plot of spleen and lung B cell helper subclusters.

We then used the scTCR-seq data of these cells to evaluate the clonal relationships between Tph, Tfh, and other cell states across tissues. Among all T cells, CTL clusters exhibited the highest degree of clonality in both the spleen and lung (Figure 7A). Quantification of TCR clonal sharing across clusters showed that B cell-helper clusters 2 and 4 in the spleen and lung showed the highest amount of sharing, indicating a developmental link between these two clusters (Figure 7B). To determine how transcriptionally defined Tph and Tfh cells from spleen and lung related to other clusters, we visualised and quantified how TCRs from each subcluster mapped across the broader phenotypic clusters (Figure 7C–E). TCRs of splenic Tfh cells were frequently shared with those of CTL clusters in the spleen, as well as lung B cell-helper clusters (Figure 7D). Splenic Tph cells showed more clonal sharing with lung B cell-helper clusters, consistent with migration of these cells from spleen to lung. Tfh cells in the lung had little overlap with any other cluster. In contrast, lung Tph cells showed clear links to splenic B cell-helper T cells and also to lung CTL cells (Figure 7E).

We next analysed TCR sharing specifically between the B cell-helper populations in the spleen and lung (Figure 7F). Splenic Tfh cells mostly shared TCRs with other splenic subclusters, suggesting limited Tfh cell migration to the lung. Splenic Tph and Tfh cell TCRs were highly overlapping (Figure 7G); splenic Tph TCRs were also highly overlapping with lung Tph cells, indicating migratory and/or developmental overlap. Th1 cells in the spleen and lung shared a distinct overlap pattern, suggesting a developmental trajectory separate from Tfh/Tph cells.

### Absence of Bcl6 expression reduces accumulation of both Tph and Tfh cells

**Extended Data Figure 8.**
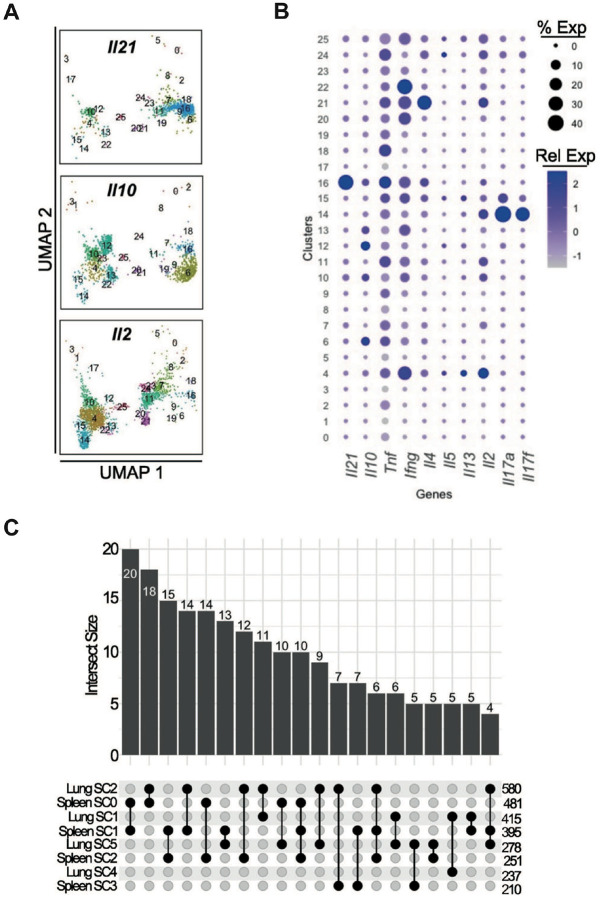
Cytokine expression across CD4 T cell clusters in CD4 T cells from Bcl6-sufficient or deficient mice **A**. FeaturePlot expression of *Il21*, *Il10* and *Il2*. **B**. DotPlot of several different cytokines representative of different T cell functional states across all clusters, relative expression shown. C. Upset plot of TCR sharing between spleen cluster 16 subclusters and lung cluster 10 subclusters. Top 20 interections are shown.

**Figure 8.**
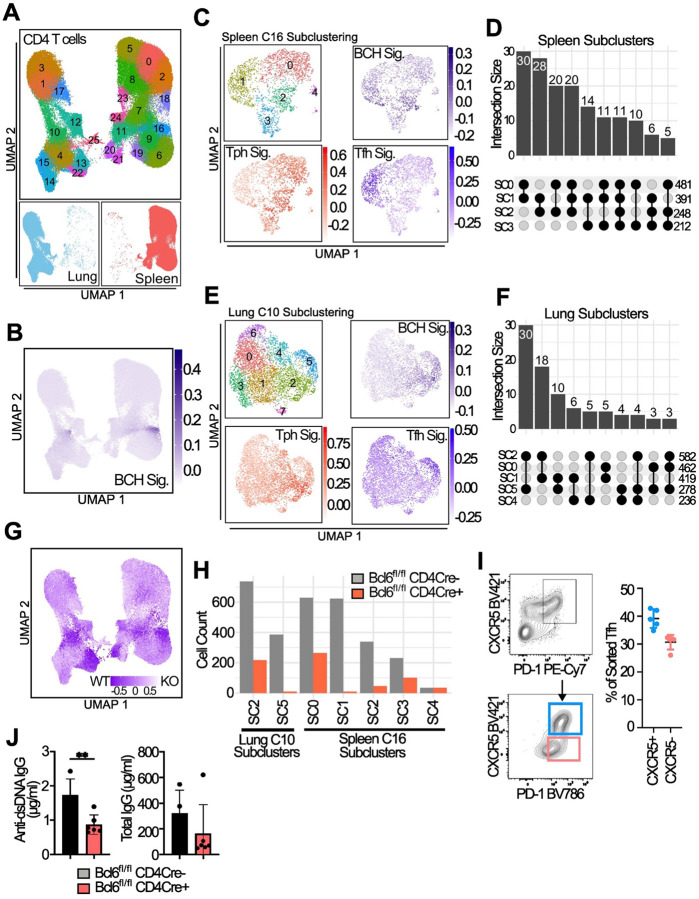
Bcl6 is necessary for B cell helper T cell development **A**. UMAP plot of CD4 T cells (both spleen and lung) from 3 month treated Bcl6^fl/fl^ CD4Cre−/+ mice. **B**. Module scoring of clusters with B cell helper signature (BCH) derived from 12-month scRNA-seq dataset using genes upregulated in spleen cluster 4. **C**. Subclustering analysis of cluster 16 from spleen showing UMAPs of: subclusters (top left), BCH signature (top right), Tph specific signature (bottom right) and Tfh specific signature (bottom left). **D**. Upset plot of TCR sharing between spleen cluster 16 subclusters. **E and F**. Similar analysis of lung cluster 10, as shown in C and D. **G**. CNA analysis showing cellular representation from either WT (CD4Cre-) or KO (CD4Cre+) cells across the UMAP. Areas of genotype overrepresentation are colour coded as displayed by the inset legend. **H**. Cell counts of Spleen and lung subclusters representing Tph and Tfh subsets, stratified by genotype. **I**. Sorted Tfh cells stimulated in vitro for 5 days and analysed by flow cytometry for expression of CXCR5 and PD-1. N=5. **J**. ELISA of total IgG and dsDNA-IgG in wildtype or Bcl6^fl/fl^ CD4Cre^+^ mice. Mann-Whitney U test, ** p = < 0.01, n=4–6 per group.

Given the shared transcriptomic features and clonal overlap between Tfh and Tph cells, we tested whether these cell populations share a common dependence on Bcl6, a transcription factor essential for Tfh cell differentiation and maintenance (18–20). We used a Bcl6-floxed CD4^cre^ model to delete Bcl6 from T cells in pristane-treated mice and analyzed CD4 T cells collected from lung and spleen 3 months after pristane treatment by scRNA-seq (Figure 8A). Spleen cluster 16 and lung cluster 10 demonstrated high expression of a B cell-helper signature, which was derived from cluster 2 of 12 month pristane-treated mice) (Figure 8B). These clusters also showed selective expression of *Il21*, consistent with B cell-helper T cells (Extended Data Figure 8A–B). Subclustering of spleen cluster 16 yielded 5 subclusters all with broad enrichment of the B cell-helper signature (Figure 8C). However, using a gene signature derived from differential gene expression analysis of Tfh vs Tph splenic subclusters in 12-month pristane treated mice, we observed enrichment for a Tfh signature within spleen SC1; a Tph signature was highly enriched in subclusters 0 and 2 (Spl SC0, SC2). Comparison of TCR sharing of spleen subclusters showed the highest levels of overlap between SC0, SC1, and SC2 (Figure 8D), consistent with Tfh and Tph having a shared developmental trajectory.

Within the subclustering of lung cluster 10, enrichment of the B cell helper transcriptional signature was restricted to subclusters 2 and 5 (Figure 8E), indicating that these subsets represent the B cell-helper T cells in lung. SC2 showed the strongest enrichment for a Tfh signature, while SC5 showed the strongest enrichment a Tph signatures (Figure 8E). TCR analyses indicated substantial TCR sharing between these two subclusters (Figure 8F). Further, both lung Tfh cells (SC2) and lung Tph cells (SC5) shared TCRs with spleen Tfh cells (Spleen SC1), indicating clonal connectivity between these populations across tissues (Extended Data Figure 8C).

CNA analysis comparing CD4 T cells from Bcl6^+/+^ or Bcl6^−/−^ cells revealed a prominent loss of B cell-helper T cells from both spleen and lung (spleen cluster 16, lung cluster 10) (Figure 8G). Quantification of cell numbers within the relevant B cell helper subclusters, encompassing both Tfh and Tph populations, demonstrated a marked reduction of both Tph and Tfh cell population in Bcl6^−/−^ mice across both compartments (Figure 8H). To further explore phenotypic plasticity, we activated sorted Tfh cells from SLE-prone B6.*Sle1yaa* mice in vitro and observed a reduction in CXCR5 expression, while PD-1 expression was maintained (Figure 8I), suggesting a partial phenotypic shift toward a Tph-like phenotype after stimulation in vitro. Together, these findings indicate that Bcl6 is required for the accumulation of both Tfh and Tph cells, and that a shared Bcl6-dependent precursor or transcriptional program may underlie their differentiation. Consistent with reduction in these populations, loss of Bcl6 expression caused a significant decrease in anti-dsDNA IgG production, along with a non-significant decrease in total IgG production (Figure 8J).

## Discussion

We performed a detailed longitudinal analyses of Tph and Tfh cells from the circulation of patients with early SLE and from pristane-induced murine lupus, in both cases demonstrating the shared transcriptomic and developmental features of these cell populations and their dynamics over time. Tph and Tfh cells from SLE patients show substantial overlap in their TCRs at multiple timepoints, consistent with past analyses of similarly sorted T cells from lymph nodes of patients with HIV (35) and type-1 diabetes (36). Our scRNA-seq analyses of SLE T cell populations highlighted a specific cluster of PD-1^hi^ T cells with features activation and proliferation that showed the most clonal sharing among sorted Tph and Tfh cells, and trajectory analyses suggest that cells may transition from a Tfh phenotype to a terminal Tph phenotype. Expanded clones of both Tph and Tfh cells could be detected at multiple timepoints over 1 year, suggesting that pathologically expanded Tph and Tfh cell clones persist systemically in SLE patients. This persistence of clonally expanded Tph and Tfh cells may impose barriers to induction of stable tolerance by either traditional immunosuppressive agents or by B cell depletion.

Tph cells, as originally described in RA synovial tissue, expressed transcriptomic, cytometric, and functional features similar to Tfh cells, with a capacity to stimulate B cell differentiation in an IL-21-dependent manner (3). Cells with similar transcriptomic, cytometric, and functional features to RA synovial T cells can be found in the circulation as well (37), and many studies have reported quantification circulating Tph and Tfh cells in autoimmune disease patient cohorts (38), yet identifying Tph cells in the circulation has been hampered by the difficulty in discriminating Tph cells from other activated T cell populations (38). Here, the recognition via scRNA-seq of a specific cluster of T cells with transcriptional features of activation, present in both sorted Tph and Tfh cell populations and showing shared clonality, provides a valuable tool in identifying circulating B cell-helper T cells and distinguishing these cells from PD-1^+^ cytotoxic T cells. The C5 cluster exhibited the highest expression of a Tph/Tfh signature score derived from kidney Tph/Tfh cells, along with high expression of *TOX*, *MKI67*, *ICOS*, *CXCL13*, and *IL21*, which strongly resembles Tph cells in the RA joints (3). Cluster C5 cell abundance correlated with both IGHV4–34^+^ plasmablasts and Ki67^+^ plasmablasts in SLE patients, suggesting that that these T cells likely provide help to B cells. These findings align with previous observations that ICOS expression on PD-1^hi^ T cells enriches for B cell-helper function (39). C5 cluster cells also express TOX, a transcription factor that promotes exhaustion and PD-1 expression (40, 41). Upregulation of TOX may suggest chronic antigen stimulation in these cells.

Parallel scRNA-seq analyses of CD4 T cells from murine pristane-induced lupus model demonstrated heterogenous populations of T cells that express features associated with B cell help, including Tfh cells as well as cells comparable to Tph cells, with strong TCR overlap between the populations in both spleen and lung. The varied B cell-helper T cell populations observed in this model are consistent with prior reports indicating a range of phenotypes of B cell-helper T cells that differ from prototypical Bcl6^hi^ CXCR5^hi^ Tfh cells. This includes CXCR4^+^ extrafollicular helper T cells in murine lupus (42), as well as CXCR5^−^ Tfh-like cells in lungs of mice with chronic Ag-driven inflammation (43), and T resident helper cells observed in lungs of mice with influenza (44, 45). Tox2, a factor related to Tox has been reported to alter the chromatin accessibility at the Bcl6 locus in Tfh cells (46).

The factors that control Tph cell differentiation are still being delineated (47). Compared to Tfh cells, Tph cells exhibit lower BCL6 expression (3), suggesting that BCL6 may not play a critical role in Tph cells. However, we find that absence of Bcl6 in T cells in pristane-treated mice substantially reduced Tph cell accumulation, particularly in spleen. Bcl6 is a repressive transcription factor that inhibits the expression of master regulators such as Tbx21, Gata3, and Rorc, which are responsible for the differentiation of Th1, Th2, and Th17 cells. Simultaneously, Bcl6 represses Id2, Runx2, Runx3, Klf2, and Prdm1, thereby promoting the expression of Tfh-related genes such as Cxcr5, Icos, Pdcd1, and Il21 (48, 49). In humans, BCL6 is expressed at high levels in germinal center-Tfh (GC-Tfh) cells compared to PD-1^low^ cells or pre-Tfh cells (50). In studies that defined the central role of Bcl6 for Tfh cells, the impact of Bcl6 loss was quite specific to Tfh cells, with limited effects on non-Tfh cells noted (18–20). Given the clonal relationship between Tfh and Tph cells, it is possible that one major path to accumulation of Tph cells occurs through conversion of some Tfh cells into Tph cells, which would explain the loss of Tph cells in the absence of Bcl6. Consistent with this, in patients newly diagnosed with SLE, CXCR5^−^ PD-1^hi^ Tph cells were persistently expanded over at least 1 year, whereas CXCR5^+^ PD-1^hi^ Tfh cells decreased over time, suggesting a potential transition of Tfh cells into Tph cells during disease progression (12). Moreover, a previous study demonstrated that the TCR repertoire overlap was observed between Tph cells and Tfh cells, and Tph cells were depleted by a Bcl6 inhibitor along with Tfh cells in the NOD.Aire^GW/^ neuropathy murine model (51). Alternatively, it is possible that Tph cells can also differentiate independently of Tfh cells, yet in a Bcl6-dependent manner. Bcl6 expression occurs transiently in T cells during the early stages of activation (52); thus, it is possible that early Bcl6 activity is required to generate a Tph cell, perhaps in a shared Tfh/Tph-precursor T cell. This would be consistent with Bcl6 being also necessary for the extrafollicular, T-dependent antibody responses previously observed (53). Additional studies are required to understand the extent to which these paths contribute to Tph cell accumulation in different settings.

This study is limited by the relatively small cohort of patients with early SLE analysed longitudinally and the inability to study tissue samples from patients. In addition, pristane-induced lupus is one of multiple lupus models that could be studied. In both the human and murine studies, the incomplete sampling of TCRs, assessing a small portion of the total T cells in each individual, may under-represent the extent of clonal sharing across T cell populations and timepoints.

In summary, these studies provide a delineation of the clonal dynamics of Tph and Tfh cells in the circulation of SLE patients over time and across tissues in a murine model of lupus. The persistence of clonally expanded Tph and Tfh cells may pose a substantial immunologic barrier to the induction long-lasting immune tolerance with immunosuppressive or B cell-depleting therapies. Targeting these expanded T cell clones directly may be required to fully erase the autoimmune T cell response established early on in patients with SLE.

## Methods

### Patient samples

This study was approved by the research ethics committees (BWH IRB #: 2014P002558), and informed consent was obtained from all participating patients for the collection of specimens. All SLE patients met the 1997 ACR classification criteria, the 2012 SLICC criteria, and the 2019 EULAR/ACR classification criteria (54–56). For the longitudinal SLE cohort used for TCR and BCR sequencing, 9 SLE patients were included who were within 6 months of disease diagnosis and had not received major immunosuppressive therapies (treatment with prednisone ≤10 mg and hydroxychloroquine was permitted), along with 10 non-inflammatory controls. Samples from the 9 SLE patients were collected at the time of enrollment, 6 months after enrollment, and 1 year after enrollment. For the high-risk for lupus study, patients who did not initially meet any classification criteria for SLE but scored 4–9 points on the EULAR/ACR 2019 classification criteria for SLE were followed longitudinally, and 3 patients who subsequently fulfilled one of the SLE classification criteria during follow-up were studied. PBMC samples were isolated by density gradient centrifugation using Ficoll-Paque Plus (GE Healthcare) and cryopreserved in FBS with 10% DMSO via slow freezing, followed by storage in liquid nitrogen.

### Bulk TCR and BCR sequencing

Frozen PBMC samples were thawed into RPMI Medium 1640 (Life Technologies #11875–085), supplemented with 10% heat-inactivated FBS (Life Technologies #16000044). The samples were stained with flow cytometry antibodies for 30 minutes on ice. After staining, the cells were washed once in PBS with 1% BSA, passed through a 70 μm filter, and stained with 1ul of a 1:100 diluted Propidium Iodide Solution (BioLegend, #421301). 7 T cell subsets and 4 B cell subsets were directly sorted from the samples using a 5L BD FACSAria Fusion cell sorter into RLT lysis buffer kept on ice. The lysate was then frozen at −80°C until shipment to iRepertoire. Bulk TCRB sequencing data were generated from DNA, and bulk BCR-IGHV sequencing data from RNA. Each lysate was split into two samples to create duplicate data. Read count data for each sample were generated by iRepertoire, including CDR3 sequences, sequence read counts for each clone, and information on the identified V, D, and J genes. These data were subsequently loaded into R (v4.2.0) for further analysis. Non-productive sequences identified by IMGT/V-QUEST were removed, and the count data were analyzed using the Immunarch package.

### Single-Cell RNA and TCR sequencing for human samples

Frozen PBMC samples from 3 out of 9 SLE patients were thawed in RPMI Medium 1640 with 10% FBS. Cells were incubated with an Fc receptor blocking solution, Human TruStain FcX^™^ (BioLegend), in Cell Staining Buffer (BioLegend) for 10 minutes at 4°C, followed by staining with flow antibodies for 30 minutes on ice. 5 T cell subsets, from non-Treg memory cells, were sorted into RPMI Medium 1640 with 10% FBS. After washing with Cell Staining Buffer, the cells were stained with a hashtag antibody (BioLegend) for 30 minutes on ice. Cells were then washed three times with Cell Staining Buffer, and 30,000 cells from each subset/donor were mixed. Then the cells were stained with TotalSeq^™^-C Human Universal Cocktail (BioLegend) for 30 minutes on ice, washed three times, and resuspended in 0.4% BSA in PBS. After counting live cells in 0.4% BSA in PBS using Trypan blue, a total of 160,000 cells in two batches were loaded onto a Chromium Next GEM Chip G (10x Genomics). cDNA and library preparation were conducted according to the manufacturer’s protocol. mRNA libraries were sequenced using the Novaseq S4 flow cell (Illumina), and CITE-seq antibody-derived tag (ADT) libraries were sequenced using the HiSeq X Ten System (Illumina). Unique molecular identifier (UMI) counts for mRNA and ADT were quantified using Cell Ranger v7.1.1. FASTQ files were aligned to the GRCh38 human reference genome using Cell Ranger v7.1.1 (10x Genomics), and gene and ADT reads were quantified simultaneously using the Cell Ranger count.

The files were then processed with the Seurat R package (v5.1.0) (57). Expression of each hashing antibody was normalized using the centered-log ratio (CLR) method, followed by demultiplexing with the HTODemux function and a positive quantile of 0.99. Doublets and cells without hashtag staining were excluded. For quality control, we removed cells expressing fewer than 200 genes, more than 3,000 genes, or containing more than 10% of total UMIs associated with mitochondrial genes. After QC, mRNA expression was log-normalized (scale.factor = 10,000), and protein expression was normalized with CLR. The top 2,000 most highly variable genes, selected through variance stabilizing transformation, were scaled. Next, 20 principal components (PCs) were calculated based on mRNA data, and samples were batch-corrected using the Harmony R package. UMAP visualization and clustering (resolution = 0.5) were performed. Tissue Tph/Tfh signatures were calculated using the differentially expressed genes (DEGs) of cluster 4 in lupus kidney single-cell RNA-seq data with the AddModuleScore function in Seurat.

Sequencing reads for the TCR library were processed through the Cell Ranger workflow (v7.1.1). Reads were aligned to a TCR reference (vdj-GRCh38), productive contigs were filtered, and CDR3 sequences were identified for each cell. Cells with matching both CDR3alpha and CDR3beta were grouped into the same clonotype. Each cell’s TCR was then paired with its transcriptomic data by matching cell barcodes. These files were then analyzed using the scRepertoire package (58).

### Mass cytometry

Cryopreserved PBMC were thawed into RPMI Medium 1640 (Life Technologies #11875–085) supplemented with 5% heat-inactivated fetal bovine serum (Life Technologies #16000044), 1 mM GlutaMAX (Life Technologies #35050079), antibiotic-antimycotic (Life Technologies #15240062), 2 mM MEM non-essential amino acids (Life Technologies #11140050), 10 mM HEPES (Life Technologies #15630080), 2.5 × 10^−5^ M 2-mercaptoethanol (Sigma-Aldrich #M3148), 20 units/mL sodium heparin (Sigma-Aldrich #H3393), and 25 units/mL benzonase nuclease (Sigma-Aldrich #E1014). Cells were counted and 0.5–1×10^6^ cells from each sample were transferred to a polypropylene plate for staining. The samples were spun down and aspirated. 5 μM of cisplatin viability staining reagent (Fluidigm #201064) was added for two minutes and then diluted with culture media. After centrifugation, Human TruStain FcX Fc receptor blocking reagent (BioLegend #422302) was used at a 1:100 dilution in PBS with 2.5 g bovine serum albumin (Sigma Aldrich #A3059) and 100 mg of sodium azide (Sigma Aldrich #71289) for 10 minutes followed by incubation with conjugated surface antibodies for 30 minutes. All antibodies were obtained from the Harvard Medical Area CyTOF Antibody Resource and Core (Boston, MA). 16% stock paraformaldehyde (Fisher Scientific #O4042–500) dissolved in PBS was used at a final concentration of 4% formaldehyde for 10 minutes in order to fix the samples before permeabilization with the FoxP3/Transcription Factor Staining Buffer Set (ThermoFisher Scientific #00-5523-00). The samples were incubated with SCN-EDTA coupled palladium based barcoding reagents for 15 minutes and then combined into a single sample. Conjugated intracellular antibodies were added into each tube and incubated for 30 minutes. Cells were then fixed with 1.6% formaldehyde for 10 minutes. DNA was labelled for 20 minutes with an 18.75 μM iridium intercalator solution (Fluidigm #201192B). Samples were subsequently washed and reconstituted in Milli-Q filtered distilled water in the presence of EQ Four Element Calibration beads (Fluidigm #201078) at a final concentration of 1×10^6^ cells/mL. Samples were acquired on a Helios CyTOF Mass Cytometer (Fluidigm). Cytometry data were normalized and debarcoded. Live cells were gated as DNA^+^ Live^+^ Beads^−^ using FlowJo (BD). Batch-corrected total live cells, were then analyzed using the Seurat R package (v5.1.0) (57) for PCA, neighborhood analysis, and clustering visualized by UMAP. Cells associated with SLE were identified using CNA (28).

### Mice and injection protocol

C57BL/6J, Bcl6^fl/fl^, CD4cre, and B6.Cg-Sle1NZM2410/Aeg Yaa/DcrJ mice were purchased from Jackson Laboratory. To generate Bcl6^fl/fl^ CD4cre heterozygotes, Bcl6^fl^ mice were crossed to homozygosity before being were crossed with CD4Cre hemizygous mice. Cre +ve and -ve, sex-matched, littermate controls were used for experiments. Pristane-induced lupus was initiated by a one-time, 0.5ml injection of Pristane (Sigma). Mice were left for stated experimental time-frames and monitored weekly. Animal protocols used in this study were approved by Brigham and Women’s Hospital Institutional Animal Care and Use Committee (protocol# 2018N000053).

### Mouse Cell sorting and sequencing

Mice were sacrificed and tissues were processed immediately for flow staining and sorting. In brief, spleen, lymph node, granuloma and lung were harvested from mice and mechanically dissociated to form single cell suspensions. Lung tissue was further treated with 100 ug/ml liberase TL and 100 ug/ml DNase in RPMI (both Sigma) for 30 minutes at 37 degrees in a shaker. Digestion was quenched with excess media, cells were washed, strained and then incubated for a further 20 minutes at 37 degrees to allow re-upregulation of Cxcr5. All cells were filtered through a 70 micron filter prior to flow staining or hashing protocols.

Resuspended cells from the spleen and lung tissues were first incubated with Live/Dead Aqua (Thermo) and FcR block (Biolegend) in PBS for 10 mins at 4°C. Cells were then stained by standard protocols with hashing antibodies (Biolegend), cite-seq antibodies: ICOS, IA/IE, CD200, CXCR6, CCR2, and CX3CR1 (Biolegend), and a staining cocktail: CD45.2 PerCP-Cy5.5, B220 BV785, CD4 PE-Cy7, TCRb FITC, CD44 e450, CD62L A780, CD25 A700, IL7Ra BV650, PD1 APC and CXCR5 PE (Biolegend). For bulk-sequencing, 2000 cells were sorted based on the gating outlined in Figure 5 directly into TCL lysis buffer (Qiagen) with 1% bME (Sigma). Lysates were vortexed to ensure proper lysis and snap frozen on dry ice. Lysates were sequenced using SMART-Seq2 RNA-sequencing (Broad Institute). Raw fastq files were processed, mapped and reads quantified using Salmon. Further downstream analysis was carried out in R. For single cell sequencing, total CD4 T cells were sorted from either spleen or lung tissue into tubes containing 0.4% BSA-PBS. For both experiments equal numbers of cells from each mouse and/or genotype were sorted, counted, pooled and processed for 10X Chromium sequencing (Centre for Cellular Profiling BWH). Approximately 30K cells were used for the 12mo experiment (n = 5 mice), and 100K cells were used for the Bcl6^fl/fl^ CD4cre−/+ experiment (n= 5 mice/group). Raw files were aligned with the reference mouse genome. Filtered reads were used for downstream analyses.

### Mouse RNA-Seq Analysis

Salmon quantification files were read into R using the “tximeta” package and a metadata table generated based on mouse donor, organ, and cell type. Raw counts and the metadata table was used by DESeq2 to normalise and filter data. Extra filters based on expression levels across multiple samples, as well as removal of mitochondrial, olfactory, and genes of unknown function. Batch effects (between mice) were removed using “Limma”. PCA was performed using prcomp and plotted with ggtools2. Differential gene expression was carried out using DESeq2 and volcano plots generated using ggplot2 also. Vst transformed data was used to plot heatmaps and carry out hierarchical clustering (pheatmap function R).

### Mouse scRNA-seq Analysis

Raw reads generated from Cell Ranger were loaded in R and analyzed by the Seurat R package. HTO data was added to Seuratobject and then normalized. The HTODemux function clustered cells based on the normalized data and assigned labeled cells to their original samples. Each hashtag corresponded to one sample origin. Cells classified as either ‘negative’ or ‘doublets’ were removed from downstream analysis. Low-quality cells with less than 200 genes or more than 10% mitochondrial content were removed from the dataset. Filtered cohorts included: 20,794 cells and 16,793 cells from the spleen and lung tissues of wildtype mice treaded pristane for 12 months, respectively; 33,554 cells and 24,923 cells from the spleen and lung tissues of wildtype mice treaded pristane for 3 months, respectively; 33,791 cells and 23,051 cells from the spleen and lung tissues of Bcl6fl/fl CD4cre mice treaded pristane for 3 months, respectively. Raw reads of the rest cells were first normalized and scaled with the NormalizeData and ScaleData functions in Seurat, respectively. The top 2000 variable genes were detected by the mean variability plot of the “FindVariableFeatures” function, and then PCA dimensionality reduction was performed with default parameters. Harmony was performed to correct batch effects between mice or different cohorts. Dimension reduction analysis was performed based on the top 10 to 20 harmonized principal components with a low clustering resolution. Standard plotting tools in Seurat (DimPlot, FeaturePlot, DotPlot) were used to visualise gene and cluster expression. FindMarkers was used to carry differential gene expression and this was visualised using ggplot2. Enrichment analysis was carried out using the top 100 genes up in Tph vs Tfh cells in the spleen using the ReactomePA package. AddModuleScore (part of Seurat package) was used to look for enrichment of gene signatures within clusters. The top 50 genes up in Tfh (vs Tph) or Tph (vs Tfh) in the spleen were used to generate the signature. TCR clonality analyses were performed with scRepertoire (v.1.7.2) R package. TCR sequences processed by Cell Ranger were exported and integrated with single-cell sequencing data by the combineExpression function. Cells with a single TCR α or β chain were removed from downstream analysis. Clonally expanded T cells were defined as T cells with both TCR-α and -β chains comprising identical VDJ genes and CDR3 nucleotides. The clonotype frequency was defined with default parameters: single (cell=1), small (1<cells ≤ 5), medium (5<cells ≤ 20), large (20<cells ≤ 100), and hyperexpanded (100<cells ≤ 500). The function DimPlot from the Seurat R package was used to visualize the distribution of clonotype frequency. To determine T cells from the same clone, TCR sequences of all the expanded cells in the certain cluster (clonotype frequency >1) were first extracted. The function highlightClonotypes in the scRepertoire R package was used to obtain the subset with the same TCR repertoire. Morisita plots were generated using standard functions in the scRepetoire package. The T cell clones were highlighted on the UMAP by DimPlot and ggplot2. Upset package in R was used to visualise TCR sharing intersections.

### In vitro co-culture assay to assess CXCR5 downregulation

Splenic Tfh cells were isolated from B6.*Sle1yaa* mice by magnetic isolation of CD4 T cells (Miltenyi), followed by sorting CD4+B220-/CD4+GITR-/CXCR5+PD1+ cells. These were co-cultured with autologous B cells at a 1:1 ratio in the presence of plate-bound anti-CD3 and soluble anti-IgM to provide TCR and BCR stimulation. Cultures were maintained for 5 days in complete RPMI media (RPMI 1640 supplemented with 10% FBS, 100 U/mL penicillin, 100 μg/mL streptomycin, 2 mM L-glutamine, and 50 μM β-mercaptoethanol). At the end of the culture period, cells were harvested and analyzed by flow cytometry, gated on live CD4^+^ I-A^+^ cells to assess changes in CXCR5 expression.

### ELISA

Total IgG and dsDNA-IgG ELISA (Chondrex) were carried out on serum from Bcl6^fl/fl^ CD4cre−/+ mice after 3 months of pristane treatment following the manufacturer’s instructions.

## Extended Data
